# Coumarins in Food and Methods of Their Determination

**DOI:** 10.3390/foods9050645

**Published:** 2020-05-18

**Authors:** Mirjana Lončar, Martina Jakovljević, Drago Šubarić, Martina Pavlić, Vlatka Buzjak Služek, Ines Cindrić, Maja Molnar

**Affiliations:** 1 Faculty of Food Technology Osijek, Josip Juraj Strossmayer University of Osijek, Franje Kuhača 20, 31000 Osijek, Croatia; mirjana.loncar@ptfos.hr (M.L.); mjakovljevic@ptfos.hr (M.J.); dsubaric@ptfos.hr (D.Š.); 2 Croatian Agency for Agriculture and Food, Vinkovačka cesta 63c, 31000 Osijek, Croatia; martina.pavlic@hapih.hr (M.P.); vlatka.buzjak.sluzek@hapih.hr (V.B.S.); 3 Karlovac University of Applied Sciences, Trg J. J. Strossmayera 9, 47000 Karlovac, Croatia; ines.cindric@vuka.hr

**Keywords:** coumarin, cinnamon, coumarin-containing foods, human exposure to coumarin, determination of coumarin in food

## Abstract

Coumarin is a natural product with aromatic and fragrant characteristics, widespread in the entire plant kingdom. It is found in different plant sources such as vegetables, spices, fruits, and medicinal plants including all parts of the plants—fruits, roots, stems and leaves. Coumarin is found in high concentrations in certain types of cinnamon, which is one of the most frequent sources for human exposure to this substance. However, human exposure to coumarin has not been strictly determined, since there are no systematic measurements of consumption of cinnamon-containing foods. The addition of pure coumarin to foods is not allowed, since large amounts of coumarin can be hepatotoxic. However, according to the new European aroma law, coumarin may be present in foods only naturally or as a flavoring obtained from natural raw materials (as is the case with cinnamon). In this paper, the overview of the current European regulations on coumarin levels in food is presented, along with the most common coumarin food sources, with a special emphasis on cinnamon-containing food. Human exposure to coumarins in food is also reviewed, as well as the methods for determination and separation of coumarin and its derivatives in food.

## 1. Introduction

The name ‘coumarin’ originates from a French term “Coumarou” for the Tonka bean (*Dipteryx odorata*), from which coumarin was isolated for the first time, in 1820 by Vogel [1]. Later, it was also found in many other plants, such as sweet clover, woodruff, bison grass, vanilla grass, cinnamon, as well as strawberry, black currant, apricot, and cherry [2,3]. In nature, coumarin and its derivatives can be found in a free form or conjugated with other molecules as glycosides [4]. Most of these compounds are not considered a risk to human health in the concentrations present in edible plants [5].

**Figure 1:**
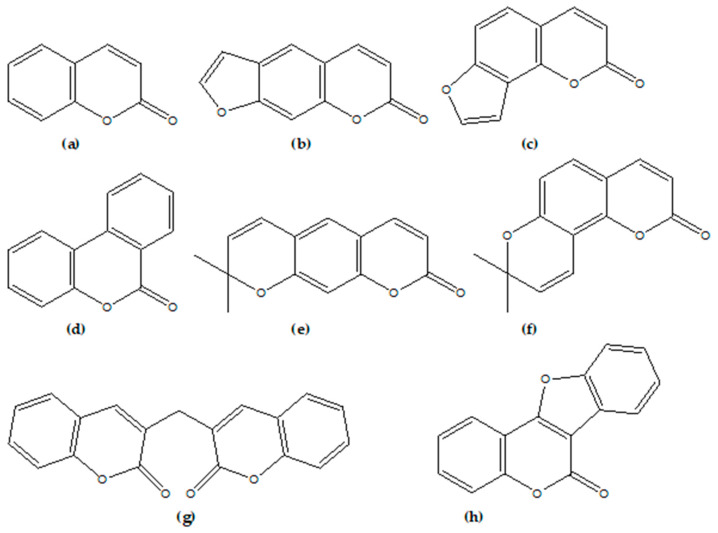
Six basic groups of natural coumarins: (**a**) simple coumarins; (**b**) furanocoumarins (linear type); (**c**) furanocoumarins (angular type); (**d**) benzocoumarins; (**e**) pyranocoumarins (linear type); (**f**) pyranocoumarins (angular type); (**g**) biscoumarins; (**h**) coumestans.

Coumarins (1,2-benzopyrones or *2H*-1-benzopyran-2-ones) represent an important family of naturally occurring benzopyrone compounds, all of which consist of a benzene ring linked to the pyrone ring [6]. Natural coumarins can be divided into six basic groups as follows: simple coumarins, furanocumarins (linear type and angular type), pyranocoumarins (linear type and angular type), bisoumarins, benzocoumarins and coumestans (Figure 1) [1].

Coumarins are considered as secondary plant metabolites that protect the plant from infections, with an important role in plant biochemistry and physiology; they act as antioxidants, enzyme inhibitors and precursors of toxic substances. Particularly, these compounds are involved in the activity of plant growth hormones and growth regulators, respiration control and photosynthesis [1].

Coumarin and its derivatives possess a wide range of biological properties that primarily depend on their chemical structure [6]. For this reason, they have been applied in a wide range of pharmacological applications such as antimicrobial agents [7], antioxidants [8,9], anti-inflammatories [10,11], anti-HIV agents [11], anticancer agents [12], anticoagulants [13], antiviral agents [14], antituberculosis agents [15]. Therefore, numerous different coumarin properties have led to their application in drug production, cosmetics, agrochemicals, optical brighteners, dispersed fluorescent and laser dyes, as well as in the food industry [16].

Coumarin is characterized by a sweet odor, often compared to the scent of fresh hay, woodruff, or vanilla [17]. Due to its recognizable, pleasant odor and the possibility to act as a fixative and enhancing agent in perfume, coumarins have been used in the perfume industry since 1882 [1,18]. In the literature, coumarin aroma has been often described as a sweet-scented a creamy vanilla bean aroma with heavy nut-like tones, but not sharp or brilliant [19]. Coumarin has also been used as a fragrance substance in other cosmetic preparations, such as shower gels, shampoo, toothpaste, toilet soaps, intimate soaps, shaving foams, body creams, face creams, hand creams, deodorants, sunscreens, aftershaves and lipsticks [20]. In addition, it has been used in tobacco to give the product a better taste and aroma whereby increasing the attractiveness of these products [21].

After coumarin was synthesized in 1868, it was first put on the market as a flavoring substance [17,22]. However, since coumarin has been found to cause liver toxicity to rats and dogs that were fed with coumarin-containing food, the usage of coumarin as a flavoring substance has become questionable [23]. Since then, although there was no existing data on carcinogenicity and mutagenicity in humans, coumarin was withdrawn from usage in the USA in 1954, based on hepatotoxic results in rats and dogs [24]. Some later researches indicated that coumarin may be related to cancer effect, but it has not yet been confirmed if coumarin has a genotoxic effect on humans [17,25].

All collected evidence encouraged the European Commission to prescribe a maximum level of coumarin in food and beverages [26]. Regulation EC 1334/2008 [27] defines the maximum level for specified cinnamon-containing food such as desserts, traditional and/or seasonal baked goods, and breakfast cereals. Today, dietary exposure to the coumarins is quite significant because of their presence in the vegetables, fruit, seeds, nuts and other diet sources, while the greatest contribution of coumarin intake is certainly through the consumption of cinnamon and cinnamon-containing foods [28].

## 2. Regulation of Coumarin in Food

The presence of coumarin in food is regulated in the EU by the Regulation (EC) No 1334/2008 of the European Parliament and of the Council 16 December 2008 [27]. According to the Annex III of this regulation, coumarin shall not be added as a substance to the food, although the maximum level for coumarin is laid down for certain compound food to which cinnamon is added as a flavoring and/or food ingredient with flavoring properties (Table 1) [27].

Although cinnamon is the main source of the coumarin from food, a limit value for coumarin in cinnamon currently does not exists. The new European flavoring regulation does not lay down any general limit values for natural ingredients contained in herbs and spices [29]. Current limit values for coumarin have been in place since January 2011. The old European flavoring regulation of 1988 specified a stricter limit value of 2 mg per kg of food for foodstuffs generally. That strict value was laid down at the time because the development of cancer resulting from a small intake could not be ruled out. However, more recent scientific studies show that this concern was unfounded [29].

### Health-Based Guidance Value

EFSA’s Scientific Panel on Food Additives, Flavourings, Processing Aids and Materials in Contact with Food [30] were asked to review the toxicity of coumarin. The panel concluded that coumarin was not genotoxic in experimental animals and therefore a TDI (tolerable daily intake) could be derived. Established TDI, based on hepatotoxicity in a two-year dog study, was 0 - 0.1 mg coumarin/kg bw. Human data from the medicinal use of coumarin evaluated by the Federal Institute for Risk Assessment (Bundesinstitut für Risikobewertung) (BfR) [31] and identification of lowest daily dose capable of inducing liver toxicity [32] resulted in the same value for TDI as already established by EFSA (2004). 

In 2008, following the request from European Commission, EFSA concluded that the evaluation of the data, discussion papers, and assessments published since 2004 does not provide a basis for changing the TDI for coumarin of 0.1 mg/kg bw [33]. TDI set by EFSA in 2004 and confirmed by BfR in 2006 and by EFSA in 2008 was reconfirmed in 2012 by BfR based on the evaluation of the human biokinetic study by Abraham [34].

## 3. Food Sources of Coumarins

### 3.1. Natural Sources of Coumarin

Coumarins occur naturally in many plants, natural spices and foods. A large number of these compounds is present in many different herbal plants, which are commonly used as medicinal plants, aromatic plants and edible plants in human and animal nutrition (Table 2). Those coumarins are distributed differently in all parts of the plants, therefore, they can be found in plants seeds, flowers, leaves, roots, and stems. Moreover, in citrus fruits, it was found that the peel is the richest part in coumarins, considering both molecular diversity and the concentration of coumarins and furanocoumarins [35]. The research of six different citrus peel extracts (sweet orange, lemon, grapefruit, bergamot, pummelo and clementine) showed that orange and clementine contain low amounts of coumarins and furanocoumarins, whereas bergamot peel had the highest total coumarin and furanocoumarin content (Table 3) [35].

Coumarins are present in many plant families such as *Apiaceae*, *Asteraceae*, *Rosaceae*, *Rutaceae*, *Rubiaceae*, *Solanaceae*, and *Lamiaceae* [36]. Generally, their occurrence in the plants depends on environmental factors as well as the seasonal changes [28]. Some of the natural plant sources of coumarin and its derivatives are listed in Table 2.

**Table 2:** Some of the natural sources of coumarins.

Coumarin and its Derivatives	Natural Sources of Coumarin	Lierature
23 coumarin compounds	The roots of *Angelica dahurica*	[37]
9 coumarins (clauexcavatins A and B, citrusarin A, clausenidin, clausenidin methyl ether dentatin, nordentatin, clausarin and xanthyletin)	The roots of *Clausena excavate*	[38]
6 coumarins (osthol, oxypeucedanin, xanthotoxin, isoimperatorin, oxypeucedanin hydrate and meranzine hydrate)	The roots of *Ferulago subvelutina*	[39]
4 coumarins (esculetin, scopoletin, fraxetin and scopolin)	The flowers of *Bombax ceiba* L. (Family: Bombacaceae)	[40]
5 coumarins (umbelliferone, scopoletin, repensin B, daphnoretin and daphnorin)	The flowers of *Trifolium repens*	[41]
12 coumarins (skimin, scopolin, scopoletin, umbelliferone, 6,7-dimethoxycoumarin, coumarin, psoralen, xanthotoxin, 5,7-dimethoxycoumarin, pimpinellin, imperatorin and osthole)	The leaves of Bamboo plants	[42]
9 terpenylated coumarins	The leaves of *Zanthoxylum schinifolium*	[43]
Coumarin glycosides and aglycone	The leaves of *Matricaria chamomilla* L	[36]
6 coumarins	The leaves of *Calophyllum inophyllum*	[44]
Coumarin, *o*-coumaric acid glucoside	The leaves of *Melittis melissophyllum* (bastard balm)	[18]
Sesquiterpene coumarins	The seeds of *Ferula sinkiangensis*	[45]
Mammea coumarins	The leaves of the tropical tree *Calophyllum brasiliense*	[46]
19 coumarins	The leaves and stems of *Murraya paniculata* (L.) Jack.	[47]
7 new terpenylated coumarins and other coumarins	The root bark of *Ailanthus altissima* (Mill.) Swingle	[48]
6 compounds, including coumarins and furanocoumarins (5-geranyloxy-7-methoxycoumarin, limettin, isopimpinellin, bergaptene, bergamottin and oxypeucedanin hydrate)	The peel of citrus grown in Colombia (Tahitian and Key lime)	[49]
2 coumarins (isomeranzin and osthole)	A sweet orange (*C. sinensis*)	[50]
Coumarins and furanocoumarins	6 citrus peel extracts (sweet orange, lemon, grapefruit, bergamot, pummelo, and clementine)	[35]

More than 1800 different natural coumarins have been revealed, mostly in plants, although coumarins have also been found in microorganisms. Some important coumarin members belonging to microbial sources are novobiocin and coumermycin from *Streptomyces*, and aflatoxins from *Aspergillus* species [28]. The presence of coumarin derivatives was also confirmed in some essential oils that are used as flavoring substances, such as Chinese cinnamon oil, cinnamon bark oil and lavender oil [51].

Epidemiological research has shown that consuming a large number of fruits, vegetables and whole grains is beneficial to human health, which at the same time can contribute to a higher intake of coumarin compounds [52,53].

### 3.2. Coumarin in Foodstuffs

Besides fruits and vegetables, some widely used dietary products such as oils (olive, soy, peanuts corn), coffee, nuts, wine, and tea also contain coumarin [54,55]. It was reported that coumarin contributes to the sweet odor of Japanese and Chinese green tea [4]. Coumarin is also present in beverages, but in very small quantities [56]. Moreover, they are regarded as significant constituents of propolis that contribute to its pharmacologically properties [57].

The maximum coumarin levels in different food are listed above, while some coumarin-containing foodstuffs that are usually used in human diet are listed in Table 3.

**Table 3:** Coumarin-containing foods used in human diet.

Sample	Detected Coumarin Level	Literature
Japanese green tea	0.26 to 0.88 µg/g dried tea	[4]
Herbal tea samples (*Melilotus Officinalis*)	Coumarin: 3.7 to 3.9 mg/L	[58]
	4-hydroxycoumarin: 111.1 µg/Lto 201.2 µg/L	
	Dicoumarol: 80.1 to 138.8 µg/L	
Propolis and propolis products	Umbelliferone: 0.05 to 1.2 µg/g	[57]
	4-methylumbelliferone: 0.05 to 1.7 µg/g	
	Scoparone: 0.45 to 7.5 µg/g	
24 vanilla extract products	Negative for coumarin	[59]
Lavender and lavandin honey	Lavandin honey(*Lavandula angustifolia x latifolia*): 100.35 µg/kg	[60]
	Lavander honey (*Lavandula latifolia*): 142.14 µg/kg	
Orange (peel)	Coumarins: 0.77 mg/kg	[35]
	Furanocumarins: in traces	
Clementine (peel)	Coumarins: 2.15 mg/kg	
	Furanocumarins: 3.66 mg/kg	
Lemon (peel)	Coumarins: 34.25 mg/kg	
	Furanocumarins: 85.43 mg/kg	
Grapefruit (peel)	Coumarins: 137.19 mg/kg	
	Furanocumarins: 267.48 mg/kg	
Bergamot (peel)	Coumarins: 131.06 mg/kg	
	Furanocumarins: 648.64 mg/kg	
Pummelo (peel)	Coumarins: 83.28 mg/kg	
	Furanocumarins: 216.05 mg/kg	
Olive oil	Umbelliferone:6.60 ± 0.32 µg/mL	[54]
	7-isopentenyloxycoumarin:1.82 ± 0.24 µg/mL	
	Auraptene: 1.82 ± 0.12µg/mL	
Soy oil	Umbelliferone: 21.00 ± 0.64 µg/mL	
	7-isopentenyloxycoumarin: 12.92 ± 0.44 µg/mL	
	Auraptene: 14.82 ± 0.55 µg/mL	
Peanuts oil	Umbelliferone: 6.63 ± 0.21 µg/mL	
	7-isopentenyloxycoumarin: 1.20 ± 0.08 µg/mL	
	Auraptene: 0.98 ± 0.04 µg/mL	
Corn oil	Umbelliferone: 8.27 ± 0.23 µg/mL	
	7-isopentenyloxycoumarin: 2.73 ± 0.09µg/mL	
	Auraptene: 0.06 ± 0.01µg/mL	

Although coumarin has been widely detected in many plants, its presence in tea leaves is poorly investigated [4]. Yang et al. [4] found that the stem part of the green tea contained much lower coumarin content than the leaf, where the most coumarins occurred in its free form. They also reported that some coumarin precursors, such as primeveroside, may be found in tea leaves. Moreover, they pointed out that the steaming time and drying temperature have an effect on coumarin content in the final green tea product. Detected coumarin content in green tea samples was much lower than the maximum permitted content which refers to the coumarin from dietary sources (Table 3).

Vanilla extract is the most common form of vanilla used today. However, due to the high cost of authentic vanilla extracts, they are usually replaced by artificial vanilla flavorings that contain a synthetically produced vanillin and/or ethyl vanillin. Due to its recognizable sharp, sweet flavor, and its possibility to boost vanilla flavor, coumarin has also been used in the production of artificial vanilla extracts. In order to examine the amount of vanillin, ethyl vanillin and coumarin, 24 vanilla extract products available on Mexican market were tested by de Jager et al. [59]. However, this research showed that coumarin was not present in any of the analyzed vanilla products, as it was often assumed (Table 3).

Recent chemical and pharmaceutical research has shown that coumarins can be considered significant constituents of propolis. Hroboňová et al. [57] investigated the presence of simple coumarins such as esculin, daphnetin, fraxetin, umbelliferone, 4-methylumbelliferone, 4-hydroxycoumarin, scoparone, coumarin, herniarin and cinnamyl alcohol in propolis samples collected from different regions of Slovakia. As a result, small differences of coumarin content in the propolis samples were observed (Table 3), and it was attributed to the different climatic and geographical characteristics, the origin of the samples, flora species surrounding the hive, bee species and other factors.

A significant source of coumarin in human diets is alcoholic drinks. *Hierochloe odorata*, for example, is used to flavor a special kind of vodka, which is made mainly in Eastern Europe, as well as *Asperula odorata* in “Maiwein“ [19,51]. Today, the dietary exposure to coumarins is quite significant, due to their presence in many fruits, plants, vegetables, seeds and other dietary sources, while, however, the greatest intake of coumarin is surely through the cinnamon-containing foods [61].

### 3.3. Cinnamon

Cinnamon has been known from ancient times as one of the most significant products, which is used in the food and beverage industry, due to its culinary and medicinal properties [62]. Because of his delicate aroma, sweet-scented and spicy flavor, cinnamon has been widely used in the preparation of desserts, biscuits, cakes, chocolates, collection of candies and other spicy confectionery, hot cocoa, tea, beverages, in decorating, and in flavoring dishes such as meat, fish, sauces [61,62]. Additionally, due to its refreshful properties and possibility to remove bad breath, cinnamon is also used as a flavoring agent in chewing gums or toothpaste [62].

Cinnamon is produced from several tropical evergreen trees of the genus *Cinnamomum*, from the dried central part of the bark, but it is worth mentioning that almost every part of the cinnamon tree including the bark, leaves, flowers, fruits and roots can be used in the medicinal or culinary purposes [24]. It was discovered by the Portuguese in the 16th century in Sri Lanka, and during 16th and 17th century, cinnamon was continuously imported to Europe. The main compounds in the cinnamon are cinnamic acid, cinnamaldehyde and coumarin, while cinnamaldehyde is the major flavor constituent in all *Cinnamomum* species [5]. Some conditions such as botanical sort, climate, as well as the collection and production conditions, strongly influence the concentration of the cinnamon compounds, and consequently the cinnamon quality and price [24].

Cinnamon is characterized by a great number of vitamins and minerals as well as the bioactive compounds, whereby polyphenols and cinnamaldehyde are the most common ones. Their amount is variable, depending on the number of different factors, such as species, soil properties, part of the plant, growth and drying conditions, harvesting times, environmental and geographic conditions, as well as extraction and analysis methods [62].

According to the plant place of origin, there are different types of cinnamon (about 250 species of cinnamon have been known) [62]. However, there are four cinnamon species widely used to obtain the spice cinnamon: Ceylon cinnamon, also known as “true cinnamon” or” Mexican cinnamon“ from the genus *Cinnamomum verum*, a native from Sri Lanka; Cassia cinnamon or Chinesse cinnamon from China (from the genus *Cinnamomum cassia*); Indonesian cassia originating from Sumatra and Java regions (*Cinnamomum burmannii*), and Vietnamese cinnamon native from Vietnam (*Cinnamomum loureiroi*). Cinnamon can be added to the food as a whole or as minced, also as extracts or oils which are produced from leaves or bark of cinnamon [62]. Today, cinnamon has been used in the flavoring, medicine and perfume industry where it is commonly used in the form of essential oil.

Ceylon and Cassia cinnamon are mostly utilized as the spices and they are available in the USA and on European markets [24]. Due to the different chemical composition of those two cinnamon species, Cassia has a stronger flavor than “true“ cinnamon and that is how it is possible to differentiate one variety from another. They also have a different coumarin concentration and Cassia is the type that contains higher coumarin content compared to the “true” cinnamon [22,61]. In comparison, in Cassia cinnamon, coumarin content is up to 1%, whereas in Ceylon cinnamon is very low (trace), about 0.004% [24]. Apart from the high coumarin level, the amount of cinnamaldehyde in Cassia is higher than in Cylon cinnamon [22]. Nevertheless, cinnamaldehyde content cannot be the only parameter according to which Ceylon cinnamon and Cassia cinnamon can be differentiated [61]. Taking the price into consideration, Cylon cinnamon has a higher price in the market compared to the Cassia cinnamon. Therefore, Cassia cinnamon is usually used on the European food market for the preparation of various kinds of food, although in some countries the usage of Cassia instead of Ceylon cinnamon is forbidden or restricted [24]. On the German sell market, Cassia cinnamon is accessible in most of the cases, although the botanical species is not marked on the packing [22].

Overall, due to the high coumarin concentration in Cassia cinnamon, human exposure to the coumarin from foods is mainly caused by Cassia cinnamon. Some data have shown that direct addition of cinnamon to foods but also the use of cinnamon oils and extracts in the food industry, may have negative impacts that are related to the fact that cinnamon contains high levels of coumarin [63].

### 3.4. Coumarin in Cinnamon-Containing Foods

Today, many of commercially accessible foodstuffs are spiced with Cassia cinnamon, and therefore, they consequently contain coumarin. In general, cinnamon sticks and ground cinnamon contain different amounts of coumarin; cinnamon sticks usually contain a higher amount of coumarin compared to the ground cinnamon [64]. Hence, the presence of coumarin in cinnamon has caused concerns because of coumarin content in some products that can exceed allowed limits set by the European regulation [5,22]. Italian research reported that about 70% of the cinnamon-flavored foods investigated had a higher amount of coumarin than allowed [61].

In this chapter (Table 4), some cinnamon-containing foods available in different markets are described, as well as detected limit of coumarin in each analyzed sample.

**Table 4:** Coumarin content on cinnamon and cinnamon-containing foods.

Analyzed Cinnamon-Containing Sample	Detected Coumarin Level	Literature
Cassia cinnamon (powder)	1740 to 7670 mg/kg	[22]
Cassia cinnamon (sticks)	<limit of detection to 9900 mg/kg	
Cylon cinnamon (powder)	<limit of detection to 297 mg/kg	
Cylon cinnamon (sticks)	<limit of detection and 486 mg/kg	
*Cinnamoum verum* bark samples	In authentic samples: 12.3 to 143 mg/kg	[65]
	In market samples: 3462 mg/kg	
*C. verum*, authentic sample	0.017 g/kg	[5]
*C. verum* barks/U.S., commercial source	0.013 g/kg	
*C. verum* barks/Sri Lanka, commercial source	0.007 to 0.9 g/kg	
*C. burmannii,* authentic sample	2.14 g/kg	
*C. burmannii/*U.S., commercial source	3.99 to 9.30 g/kg	
*C. loureiroi,* authentic sample	6.97 g/kg	
*C. loureiroi* barks/Vietnam, commercial source	1.06 g/kg	
*C. cassia,* authentic sample	0.310 g/kg	
*C. cassia* barks/U.S., commercial source	0.085 to 0.262 g/kg	
*Cinnamomum spp.* Barks/U.S., commercial source	5.79 g/kg	
*Cinnamomum spp.* Barks/U.S., commercial source	21.0 to 41.7 g/kg	
*Cinnamomum spp.* Powder/U.S., commercial source	2.06 to 6.19 g/kg	
Cinnamon and apple sauce/local store	5.0 mg/kg	
Cinnamon pecan/local store	16.0 mg/kg	
Ice cream topper/local store	3.0 mg/kg	
Breakfast cereals/local store	4.0 to 44.0 mg/kg	
Instant oatmeal/local store	56.0 mg/kg	
Bread/local store	20 to 29 mg/kg	
Muffin and quick bread mix/local store	20.0 mg/kg	
Bun/local store	3.0 mg/kg	
Roll/local store	24 mg/kg	
Cracker/local store	9 mg/kg	
Swirl/local store	6.0 mg/kg	
Granola bar/local store	38.0 mg/kg	
Toaster pastries/local store	3.0 mg/kg	
Graham snack stick/local store	13.0 mg/kg	
Rice snack/local store	3.0 mg/kg	
Dietary supplements/commercial store	2450 to 3610 mg/kg	
Traditional and seasonal bakery	3.8 to 35 mg/kg	[64]
Breakfast cereals	0.9 to 10.0 mg/kg	
Fine bakery ware	0.4 to 53.4 mg/kg	
Desserts	1.0 to 5.1 mg/kg	
Crisp bread	16.0 to 23.0 mg/kg	
Tea with cinnamon	0 to 12 mg/kg	
Cassia Cinnamon	3612 mg/kg	[19]
Cinnamon (category not labelled)	2419 mg/kg	
Cereals and bakery products	9.0 mg/kg	
Cinnamon cookies	25.0 mg/kg	
Cinnamon-flavored Liqueur	-	
Vodka-flavored with sweet grass	4.0 mg/kg	
Mulled wine	-	
Milk-containing food (yoghurt, rice pudding, quark cheese)	0.7 mg/kg	
Biscuit	7.0 mg/kg	[61]
Teas	64 mg/kg	
Chocolate	10.0 mg/kg	
Dessert	3.0 mg/kg	
Cake	6.0 mg/kg	
Confectionery (candies, comfits, drops, chewing gums, pralines, jellies)	4.0 mg/kg	
Ginger bread	16.4 mg/kg	[63]
Bakery products	18.0 mg/kg	
Cinnamon-containing cakes	22.5 mg/kg	
Flatbread with cinnamon filling (lefse)	6.8 mg/kg	
Cinnamon stick	49.9 mg/kg	
Cinnamon powder	2350 mg/kg	
Tea with cinnamon	105.0 mg/kg	[66]
Cinnamon star cookies (‘‘Zimtsterne’’)	39.4 mg/kg	
Cereals with cinnamon	25.5 mg/kg	
Desserts with cinnamon	10.2 mg/kg	
Chocolate with cinnamon	9.4 mg/kg	
Almond cookies	16.2 mg/kg	
Mulled wine	0.2 mg/kg	
Gingerbread cake	10.3 mg/kg	
*Some Bakery products:*		[67]
Fruit loaf	6.2 mg/kg	
Apple pie	<1 mg/kg	
Carrot cake	3.7 mg/kg	
Muffins	18.2 mg/kg	
Fruit cake	3.3 mg/kg	
Breakfast Cereal/Muesli/Porridge	<1 to 38.2 mg/kg	
Cereal bar	<1 mg/kg	
*Tea/Beverages:*		
Chai tea	2.1 mg/kg	
Spiced/herb tea	1.8 mg/kg	
Nescafe cappuccino powder	<1 mg/kg	
Cappuccino/latté	1.4 mg/kg	
Chocolate drink-Nestlé Nesquik chocolate flavor	<1 mg/kg	
Ice cream/pudding	<1 to 2.0 mg/kg	
Snack (roasted peanuts)	48.5 mg/kg	
Rice	<1 to 1.2 mg/kg	
Vegetable dishes	<1 to 3.6 mg/kg	
Meat dishes	<1 to 3.6 mg/kg	
Soup/sandwich filler	<1 mg/kg	
Cooked meat	<1 mg/kg	
*Spices:*		
Mixed spice	456.0 mg/kg	
Ground cinnamon	1657.0 mg/kg	
Cinnamon stick	86.7 mg/kg	
Curry powder	51.5 mg/kg	
Moroccan spice	63.3 mg/kg	
Cooking sauces	<1 to 6.10 mg/kg	
*Infant food:*		
Heinz Breakfast Oat & Apple cereal for babies	5.55 mg/kg	
Organix infant carrot cake	10.9 mg/kg	

In order to detect the coumarin content and identify possible factors that influence the coumarin level, cinnamon samples (cylon and cassia powder and stick samples) from the German market, as well as cassia bark samples of trees imported from Indonesia were investigated (Table 4) [22]. Coumarin and other volatile constituents of cinnamon, such as cynnamaldehyde, cinnamic acid, cinnamyl alcohol and eugenol, as the major constituents that effect on cinnamon smell and taste, were investigated, as well as the safrole, which is considered as a possible contaminant in powdered spices.

The comparison between stick and powder of cassia and cylon cinnamon samples, indicated significant differences in the content of analyzed components. For instance, the content of cynnamaldehyde was higher in the cassia powder and stick samples compared to cylon samples. Moreover, the investigation of the same botanical species, detected lower concentration of cynnamaldehyde, cinnamyl alcohol and eugenol in the stick samples, whereas concentrations of cinnamic acid were higher in the powder samples [22].

A correlation among eugenol and coumarin levels was observed, where, by increasing the coumarin level, eugenol level also incerased, and vice versa. The same correlation with coumarin was noticed for cinnamyl alcohol, whereas conversely correlation was noticed with cinnamic acid and cinnamaldehyde. In cassia samples, the coumarin content was higher in the stick samples than in powder samples (Table 4). They also confirmed a large variation of coumarin level in cassia bark samples from the same packing. Although supposed to originate from the same harvest, it was noticed that cassia sticks from the same packing, had different coumarin concentrations. They also noticed a significant variation in the coumarin content in the trees, even within the bark from the same segment of a single tree, that may contain levels of coumarin ranging between undecectable and high levels [22].

Ananthakrishnan et al. [65] investigated coumarin and another phenolic level in authentic *Cinnamomum verum* bark samples, as well as samples from the Indian market (Table 4). They noticed a considerably higher amount of coumarin and cinnamaldehyde in the samples from the market that indicated a possible addition of Cassia cinnamon in those samples.

Cinnamon is also widely used in the USA to flavor various foods. Wang et al. [5] analyzed coumarin content in authentic cinnamon bark samples as well as bark and powder cinnamon samples from the local market, cinnamon-flavored foods, and cinnamon food supplements bought in the local stores in USA (Table 4). The results verified that coumarin presented only in small amount in the true cinnamon, *C. verum*, while Indonesian cinnamon, *C. burmannii* contained larger amounts. Coumarin was detected in all of the analyzed products, while their chemical composition revealed that Indonesian cassia, *C. burmannii* is the constituent part of all of the analyzed foods products.

Additionally, Ballin et al. [64] analyzed coumarin content in 74 products with cinnamon from the Danish market, acquired from local bakery stores, supermarket warehouse, and directly from importers. They classified the samples into several categories such as traditional and/or seasonal bakery ware, breakfast cereals, fine bakery ware, desserts, chrisp bread and tea (Table 4). This study showed that some bakery products with cinnamon showed a higher coumarin content than it has been prescribed by EU regulation. Possible explanations for this exceedance are poor knowledge about EU regulation and the estimation of cinnamon amounts that may be added to products to not exceed the prescribed limits. For this reason, they have also made a practical guidebook in order to help the manufactures of bakery products with cinnamon to produce the products without fear of exceeding the EU limits for coumarin.

Furthermore, Sproll et al. [19] found a significant amount of coumarin in cereals and bakery products, whereas the highest content of coumarin was found in Germany’s traditional cookies (Table 4). The same research showed that cinnamon-flavored beverages, such as liqueurs and mulled wine, did not contain coumarin levels above the detection limit, while vodka flavored with sweet-grass contained coumarin, but lower than the permitted level.

Lungarini et al. [61] analyzed coumarin and cinnamaldehyde contents in some Italian cinnamon-containing foods (Table 4). They proved that the majority of the analyzed foods (70%) exceeded the maximum level of coumarin content. The results of this study were considered by the Italian Minister of Health, and this act resulted in consumers’ better understanding of possible consumption risks of the coumarin-containing food.

In 2006, the Federal States in Germany analyzed the cinnamon samples and of the most popular cinnamon-containing foods (Table 4) [66]. In the most of tested food samples, Cassia cinnamon was the overcoming ingredient, whereas coumarin levels significantly surpassed the maximum allowable level (2 mg/kg in foodstuffs and beverages) of the EU Flavorings Directive 88/388/EEC. Thanks to that research, most of the manufacturers in Germany were compelled to reduce the coumarin level in their products during 2007 and 2008.

In order to identify the coumarin content and estimate the dietary exposure to the coumarin throughout the consumption of cinnamon-containing foods and drinks in the UK, 80 commonly-consumed foods were analyzed [67]. The highest coumarin content was identified in the ground cinnamon (1657 mg/kg) (Table 4), whereas the average dietary exposure to coumarin by the UK population was generally lower than the safety limit.

## 4. Human Exposure to Coumarin

### 4.1. Health Risk Assessment

The human exposure to coumarin is an important factor to be taken into consideration in the health risk assessment from different foods. Therefore, in the last several decades, scientific institutions have increasingly investigated the human health impacts caused by the coumarins in food. Owing to the hypothetic toxicity, quantification, and risk assessment is quite significant, especially for the children’s safety who can reach TDI much faster than adults [17].

Plants, fruits and vegetables are rich in polyphenols, including coumarins, which are antioxidants, contributing to the plant’s beneficial effects to human health. People in different countries mainly have different eating habits, including the consumption of coumarin-containing food as well, the important fact to be considered when assessing the potential risk of coumarin intake [66].

The extensive information about the routes of elimination of coumarin metabolites shows the significant variations between humans and animals [51,63]. It is concluded that coumarin metabolites in rats are removed through the feces, while in humans they are commonly excreted in the urine. Therefore, the retention time of coumarins and its metabolites is shorter in human organisms than in rats, thus, it is supposed that humans are less sensitive to the coumarin toxicity than rats [63]. However, the results showed that coumarin can cause liver toxicity in a sensitive group of humans [63], therefore, it is necessary to be very careful with the intake of coumarin-containing substances in order to avoid potential risk for consumers.

Based on the potential hepatotoxic effects of coumarin in rats and dogs, which were fed with coumarin-containing food, Lake [51] investigated the safety for humans of coumarin intake through foods and from fragrance use in cosmetic products. He took into consideration toxicity and carcinogenicity data with the estimated human exposure from food and cosmetic products, and concluded that human exposure to coumarin from these sources should pose no health risk to humans. The maximum daily intake of coumarin through the diet for a 60-kg consumer has been estimated on 0.02 mg/kg/day. However, if fragrances used in cosmetic products, whose maximum daily coumarin exposure has been estimated to 0.004 mg/kg/day, were taken into consideration, the maximum coumarin daily intake both from the diet and cosmetics could be estimated on 0.06 mg/kg/day [51]. Lake also found that coumarin metabolism and toxicity showed significant differences when humans are compared to the rodents. In most cases, coumarin is rapidly absorbed and secreted in the urine as glucuronide and sulfate conjugates in humans.

### 4.2. Risk of Intake of Cinnamon-Containing Foods

Taking into consideration that cinnamon is assumed to be the most important source of coumarin in food, it can be concluded that the greatest human exposure to coumarin is from cinnamon-containing foods or cinnamon as a spice. However, even with increased cognitions of the coumarin levels in food, it is still difficult to determine the maximum daily intake of coumarin from cinnamon and cinnamon-containing foods [17,66].

With the assumption that people usually eat a higher amount of cinnamon-flavored food during Christmas time, Germany’s Federal Institute for Risk Assessment (BfR) performed an interesting phone survey about the consumption of 10 different cinnamon-flavored foods during Christmas time by German adults [66]. During three days of the third week in December 2006, more than 1000 randomly selected people aged 14 or older were surveyed. The inquiry and report also included socio-demographic characteristics like age and gender. To evaluate the individual coumarin intake, a given consumption data was multiplied by the mean coumarin levels of the foods. The results showed that the average coumarin intake was higher in men (5.8 mg per week), than in women (5.0 mg per week). The assumption is that this may be because of the general lower food and calorie intake by women. Moreover, the results confirmed a lower consumption of cinnamon-containing foods at higher ages, while children consumed the highest amount of sweet food with cinnamon. Due to the children’s lower body weight, it was concluded that children represent a risky group that could reach TDI-limit very fast.

Furthermore, Ballin et al. [64] calculated that, if a child of 30 kg consumed 250 mL of tea with a coumarin content of 12 mg coumarin/L, it could reach the limit of TDI. However, it is important to mention that tea, as an important foodstuff in the human diet, which can contribute to the overall daily intake of coumarin, has not been incorporated in the EU regulation. Additionally, Sproll et al. [19] calculated the maximum amount of food that could reach the TDI-limit for children and for adults. They reported that children could surpass the prescribed TDI if they ate 3–4 cinnamon star cookies, whose weight is 5 g and established coumarin level of 88 mg/kg, while an adult could eat nearly 10 of the same cookies to exceed the prescribed limit. Children could also surpass the TDI if they ate a 75 g portion of breakfast cereals, while adults could exceed the TDI by consumption three portions.

Based on the investigation of coumarin in Norwegian foods, Fotland et al. [63] estimated toxicity and defined a new TDI for coumarin in the food of 0.07 mg/kg bw day. They concluded that small children could exceed the TDI if they eat their traditional meal which is oatmeal spiced with cinnamon, several times a week, while adults could exceed the TDI if they drink a tea with cinnamon and consume cinnamon food supplements. Moreover, it was found that the daily intake of 0.4 mg coumarin/kg bw for adults could be achieved through consuming four cups of tea with cinnamon. Therefore, for those who often consume this type of tea, a daily coumarin intake can be significantly increased.

### 4.3. Other Possibilities for Human Exposure to Coumarin

Other than oral exposure, dermal exposure to coumarin must be also taken into consideration [51]. Fragrances in cosmetic preparations such as antiperspirant deodorants, body lotions, bath products, perfumes, creams, shampoos, hair preparations, shower gels and toilet soaps can be a significant source of coumarin. The research that was conducted in Italy for 283 cosmetic preparations that were collected in various Italian shops showed that fragrances were present in 52.3% of products, in which the total share of coumarin was about 30% [20]. Therefore, in addition to the oral exposure, dermal coumarin exposure is also very important, but in contrast to the use in food production, there is no limitation for synthetic coumarin to be used as an ingredient in the fragrances in cosmetic products [27].

Furthermore, it was believed that cinnamon-based food supplements lower the blood sugar level as well as the blood lipid concentrations in diabetics. Within the scope of a health risk reassessment conducted in June and August 2006, Germany’s Federal Institute for Risk Assessment (BfR) showed concern that can arise for consumers by taking cinnamon-based food supplements, since these products may contain high levels of coumarin. The coumarin content in those supplements is strongly dependent on the cinnamon type and the way it is absorbed in a human organism. If Cassia cinnamon is used in the form of powder, there is a possibility for the consumers, depending on the dose, to exceed the TDI of coumarin [68].

Nowadays, an estimated exposure to coumarin brings out the major uncertainties because of the lack of systematic measurements of its levels in foods. The European food industry complains that the European regulations are too rigorous and advocate that coumarin in food does not pose a risk to human health. The possibility of surpassing the TDI by consuming food exits only if the limit value of 50 mg per kg of food is reached completely and if large amounts of such foods are eaten on a daily basis. However, in fear for their market, some producers of cookies have decided to change their recipes in order to reduce the amount of coumarin in their products, whereas others are trying to achieve very low coumarin levels in their products by using various cinnamon-like aromas [61].

## 5. Determination of Coumarins in Food

### 5.1. Extraction of Coumarins

There are many techniques in the literature described for the extraction of coumarins from food (Table 5). Due to the different structures of coumarin derivatives, different solvents, as well as post-extraction isolation procedures, may be used. Generally speaking, extraction of coumarins from dry or fresh plant material, can be obtained by solvents of different polarities. Also, looking at isolation procedures, they can be crystallized by cooling or concentrating the solvent depending on their structure [69].

A systematic study of coumarin extraction carried out by Bourgaud et al. [70] showed that the extraction with ethyl acetate, diethyl ether and chloroform was not adequate and applicable in the extraction of coumarin, unlike extraction using polar solvents such as methanol, ethanol and water. In addition, the extraction time did not affect the amount of coumarin extracted, and thus the amount of coumarin did not differ significantly in the extract obtained by using Soxhlet extraction with methanol for 48 h from that obtained by stirring on a magnetic stirrer for 30 min at room temperature. Sproll et al. [19] compared the efficacy of 80% methanol, 80% ethanol, acetonitrile and chloroform for the extraction of coumarin in bakery products. This has shown that the results observed with plant material [70] can also be applied to food, since methanol is also the most effective solvent for extraction of coumarin in food. Methanol has also been shown in other studies to be the most effective solvent for the extraction of coumarin among other organic solvents [71,72]. Furthermore, the influence of agitation type, solvent concentration, sample weight and extraction time was examined to obtain the optimal amount of coumarin. With regard to the agitation type, the effect of stirring and ultrasound on the amount of coumarin from 46 samples was examined, where it has been shown that the amount of coumarin in the extract obtained by stirring was 3% higher. As shown above, time did not affect the amount of coumarin, although both examined extraction times of 10 and 30 min were relatively short. The most significant influence on the amount of extracted coumarin had the amount of sample and the concentration of the solvent, so it has been proved that 80% methanol is the most effective for extracting 10 g of sample, which was shown by applying response surface design using the central composite algorithm [19].

In this case, the extraction is referred to the solid and powder food products, since liquid products such as alcohol and juice can be injected directly into HPLC, even without dilution. For products such as milk, yogurt and other dairy products, it is necessary to dilute the products with methanol before analysis to obtain a mixture of 80% methanol-water (*v*/*v*) [19]. Nevertheless, it has been shown that for the detection and quantification of coumarin in different products, it is sufficient to carry out direct extraction with 80% methanol with the necessary homogenization of food. Using the method developed in the work by Sproll et al. [19] matrix interferences are low and emulsions are not formed, even with products containing a lot of fat. For this reason, according to Sproll et al. [19] extraction using 80% methanol was under consideration in Germany for inclusion in official methods for the preparation of samples for the determination of coumarin in food. The same was shown in the work of Rahim et al. [73], whereby solid food was extracted with methanol using ultrasound for a period of 10 min to enhance extraction. Extraction by ultrasound for 10 min was sufficient, since extending the extraction time does not increase the extraction efficiency. Soft drinks and juices were analyzed directly, without extraction and dilution, while products such as dairy products were diluted with methanol in the ratio of water:methanol (1:10) before analysis.

Before this, in a paper by Belay and Poole [74], components from different foods were extracted in different ways as shown in Table 5. In addition to methanol, ethanol and ethyl acetate were most commonly used for extraction, and for certain foods, the pH was adjusted to 2 to ensure adequate recovery of the acidic flavor compounds present in natural vanilla.

**Table 5:** Overview of extraction parameters for coumarin and related components.

Samples	Type of Extraction	Extraction Parameters				Literature
		Solvent	Temperature	Time	Solid-Solvent Ratio	
Cinnamon samples	Ultrasound assisted extraction	Methanol	-	1 h	5 mg/1 mL	[17]
Tea samples					50 mg/1 mL	
Different breakfast cereal samples					100 mg/mL 250 mg/1 mL	
Milk rice					250 mg/mL	
Cinnamon dessert					100 mg/mL	
Solid food containing coumarin	Stirring on a mechanical shaker	80% Methanol (*v*/*v*)	Room temperature	10 min	15 g/50 mL	[19]
Propolis	Maceration	Ethanol	22 °C	3 days	250 g/500 mL	[57]
Herbal tea from sweet clover plant (*Melilotus officinalis*)	Stirring	Methanol		90 min	0.1 g/15 mL	[58]
Dried stem bark powders	Soxhlet apparatus	Methanol		3 h		[65]
The seeds of *Dipteryx odorata* (tonka beans)	Soxhlet extraction	Ethanol, Methanol and acetonitrile		8 h		[71]
Cassia Bark (*Cortex Cinnamomi*)	Ultrasound assisted extraction	Methanol	Room temperature	30 min	0.5 g/25 mL	[72]
Food containing soft drinks and juice, infant formula and food, cereals, flours and snacks	Ultrasound assisted extraction	Methanol		10 min		[73]
Chocolate samples	Ultrasound assisted extraction	95% Ethanol (*v*/*v*)		15 min	5–10 g/15 mL	[74]
Vanilla-containing soft drinks	Liquid-liquid extraction H_2_SO_4_ for adjusting pH = 2	ethyl acetate			25 mL/4 × 25 mL	
Vanilla flavored ground coffee	heated under reflux	ethyl acetate		30 min	10.5 g/100 mL	
Cookies	Soxhlet apparatus Mixed with H_2_SO_4_	Chloroform		4 h	10–15 g/100 mL	
Ice cream or vanilla pudding	Ultrasound-assisted extraction H_2_SO_4_ for adjusting pH = 2	70% (*v*/*v*) ethanol		30 min	15 g/25 mL	
Chocolate milk	Liquid–liquid extraction	Chloroform			Diluted with water (1:1) 50 mL/4 × 25 mL	
Flavouring or spice	Stirring on a mechanical shaker Ultrasound-assisted extraction	70% Ethanol	30 °C	30 min 10 min	1 g/50 mL	[75]
Biscuit or chocolate					5 g/50 mL	
*Cinnamomum cassia* Blume	Ultrasound-assisted extraction	80% Methanol	-	30 min	0.5 g/20 mL	[76]
*Meliloti* herba	Stirring on a mechanical shaker	Water	22 °C	60 min	5 g/30 mL	[77]
Crude propolis		Ethanol	22 °C	72 h	1 g/40 mL	
Encapsulated Cinnamon Flavoring Powder	Ultrasound-assisted extraction	80% Methanol	-	20 min	100 mg/50 mL	[78]
Vanillin samples	Mixing	Aceton	-	-	0.251 g/5 mL	[79]
Cassia cinnamon, chamomile tea	Stirring	Water (82 °C)	23 °C	1 h	1 g/12 mL	[80]

### 5.2. Methods for Determination of Coumarins

Given the presence of coumarins and coumarin derivatives in numerous plants, and consequently, in food, many researchers have developed methods for the detection and quantification of coumarin and coumarin derivatives. Early methods for determining coumarin included paper chromatography, thin-layer chromatography, as well as colorimetric assays and polarography, but today, high-performance liquid chromatography (HPLC) is the most commonly used method [19]. The polarographic method for the determination of coumarin in sweet clover herbage was developed in 1988 [75]. Coumarin was extracted with boiling ethanol, which was evaporated to 1 mL, and then ethanol and a solution of tetraethylammonium iodide were added to the sample before measurement. Polarographic measurement was performed using a dropping mercury electrode.

In newer literature, several different methods are proposed for the determination of coumarin, including gas chromatography-mass spectrometry (GC-MS), high-performance liquid chromatography (HPLC) with UV and fluorescence as well as mass spectrometry. All of these methods have their advantages and limitations, which most often include the financial cost of expensive equipment and training, as well as the environmental aspect of consuming large amounts of organic solvents and energy consumption [81]. In addition, a great problem with the simultaneous detection of a large number of coumarins is the problem with the diversity in structure and thus in the polarity of different coumarins. The detection and quantification methods used should be applicable to different samples, sensitive to low coumarin concentrations, as well as reproducible and accurate. In this section, we will list some of the methods used, along with information on detection and quantification conditions (Table 6), specific components detected, and the advantages and disadvantages of the methods, to help researchers more easily decide which method to use in their future work.

#### 5.2.1. Chromatographic Methods

Going back to the 20th century, the chromatographic methods used included paper and thin layer chromatography, most often only to detect the components present in the samples. Swain identified coumarins in 1952, using filter-paper chromatography, since it was shown that coumarins are involved in enzymatic browning in potatoes [82]. Various mobile phases, as well as reagents for spraying paper, were used to identify coumarin by making them noticeable on paper and display R_F_ (retention factor) values. R_F_ actually represents the distance traveled by the compound, divided by the distance traveled by the solvent; the number is distinctive, thus representing how the component can be identified. Among the various mobile phases used, the combinations of n-butanol-acetic acid-water and amyl alcohol-acetic acid-water proved to be the most appropriate, since the glycosides and aglycones could also be separated. The most common problems encountered with other mobile phases were elliptical and elongated spots with tailing. After developing the chromatogram, the components were detected using ultraviolet light, and various paper spraying reagents were tested. For the detection of coumarin, a paper spray prior to detection with 2N NaOH was required. Displaying the R_F_ values for all components tested, indicates the suitability of the above method for separating and identifying the coumarins and other components tested from different sources. In addition to paper chromatography, thin-layer chromatography (TLC) is also one of the earliest used chromatographic techniques. This method, in the context of coumarin determination in foods, has been most commonly used to determine vanilla flavors [74,83]. For the qualitative as well as the quantitative determination of coumarin in the vanilla flavorings, the pre-adsorbent silica gel plates were used as stationary phase, while the toluene-methanol solvent mixture (97:3, *v*/*v*) was used as the mobile phase. The plate was sprayed with NaOH and then placed under UV light to spot component stains. Coumarin was identified with respect to fluorescence, or the appearance of color, and quantified using a densitometer [83]. In addition to detecting coumarin, TLC has also been used to detect a number of components such as vanillin, ethyl vanillin, 4-hydroxy-benzaldehyde, 4-hydroxybenzoic acid, 4-hydroxybenzyl alcohol, vanillic acid, coumarin, piperonal, anisic acid and anisaldehyde in vanilla flavor as well as in foods containing vanilla flavors. To make the separation process selective and reproducible, an automated multiple development (AMD) system was used. Silica gel plates were used as a stationary phase, and to ensure good separation and resolution without tailoring of polar components, a stepwise mobile phase including chloroform, ethyl acetate, 1-propanol, acetic acid and hexane was used. By developing such a method that allowed the separation of different components from food, beverages and confectionery products, it was shown how TLC can be used in this direction and how it is a suitable technique for determining different components. The main advantages of TLC for testing the composition of vanilla extracts and vanilla-containing foods are the simplicity of sample preparation, fast analysis and a high sample throughput at reduced costs compared to the more advanced analytical techniques. Considering all the above recommendations, this method could be a suitable routine method for quality control [74]. In addition, TLC is a method that has evolved over the years, so high-performance thin layer chromatography (HPTLC) was created, which enables the automation of certain steps, increases resolution, and can make quantitative measurements more accurately. Krüger et al. [17] have determined the amount of coumarin in 43 commercially available cinnamon and cinnamon-containing foods using HPTLC and HPTLC-MS while developing a method that combines several techniques called high-pressure thin layer chromatography-fluorescence detection-mass spectrometry/effect-direct analysis (HPTLC-FLD-MS/EDA). Considering the positive characteristics of TLC, and applying a more advanced version, the idea was to develop a quicker and simpler method that would allow simultaneous screening and quantification at low concentrations for a large number of samples, which is especially important for fast and routine analysis of multiple products. The problem of the food analysis is the complexity and variability of the matrix, so the composition analysis are difficult and often involves different pretreatments. The aforementioned developed method for the analysis of various food products, such as tea, breakfast cereals, milk rice, jam, cinnamon stars and cinnamon buns, shows a high specificity for coumarin, without the first additional sample processing. The samples were extracted and diluted and then, after the completion of the chromatography process, derivatized and as such were detected using fluorescence, 17 samples at a time. In order to show the efficiency of the method, the precision and repeatability of the method was determined, even for foods such as cereals and milk rice whose matrix is complex and affects the results, precision is between 4%–7% and repeatability is below 10%. Given the low LOD (limit of detection) and LOQ, the method indicated allows coumarin in different foods to be determined at low concentrations, such as 200 pg/6-mm band and 400 pg/6-mm band, respectively. In addition, HPLTC was combined with effect-directed analysis, as a powerful bioanalytical tool, to provide additional information on bioactive components in the samples and to make a complete risk assessment [17].

Today, by using chromatography, rapid and effective methods for the detection and quantification of coumarin in various samples can be developed, such as the method by Solaiman Al-Zehouri [76], where they extracted Cinnamomum cassia Blume by ultrasound and then analyzed obtained extracts by HPLC. It is important to emphasize that despite the numerous papers, it is problematic to precisely separate the simple coumarins because of the similarity of the chemical structure as well as the polarity [77]. In China, the amount of cinnamaldehyde, cinnamic acid, cinnamyl alcohol, and coumarin were examined as important components in 44 samples of Cassia bark from fields and markets using HPLC. In addition to the quantification of the components themselves, fingerprinting was performed for five different types of collected cassia bark and it was shown that five significant peaks were sufficient to distinguish the original Cassia bark and as such could be used in quality control testing as a rapid and reliable method. The content of coumarin, depending on the type of cassia bark, ranged from 0.01–12.18 mgg^−1^ among the collected 44 samples. [72].

In addition to HPLC, UHPLC can be used to develop a shorter method with less consumption of organic solvents, but with equally good peak separation. The consumption of organic solvent is directly related to the duration of the methods that, by using UHPLC, can take several minutes compared to 10–90 min with HPLC [77].

Using UHPLC-DAD, the content of coumarin was analyzed in 74 samples of various cinnamon-containing foods on the market in Denmark [64]. Internal calibration with 4-methylumbelliferone was carried out, and this analysis confirmed that the problematic category of food was a fine bakery in which EU limit for coumarin was exceeded in almost 50% of cases. These results confirm that this method can be used for quick and routine determination of coumarins in foods. UHPLC-DAD is also a method that can also be used to determine components such as coumarin, trans-cinnamic acid, trans-cinnamaldehyde and eugenol in encapsulated cinnamon flavoring powder. This powder is actually cinnamon essential oil encapsulated with different carriers, which improves product characteristics, such as stability, oxidation, and thus ensures a longer shelf life of the product. On the other hand, this affects the way the sample is prepared so the efficiency of extraction, it this case is even more important. That is why the authors optimized the extraction process, applying different extraction parameters, to ensure maximum process efficiency. One of the important features of the UHPLC method is the shorter analysis time compared to HPLC, and therefore the method developed and validated here lasted 12 min, with concentrations of coumarin, trans-cinnamic acid, trans-cinnamaldehyde and eugenol determined in the samples [78].

Since some of the coumarins contain fluorophore in their structure it is possible to use fluorescence detectors (FL), in addition to UV and DAD detectors, which Machyňáková and Hroboňová [77], used in their work to detect coumarin derivatives and metabolites from Meliloti herb, propolis tincture and crude propolis. One of the important characteristics of coumarins for determination using UV/DAD and FL detectors is their absorbance and fluorescence in the UV range. Two different wavelengths appeared to be suitable for the determination of several coumarins, such as 4-hydroxycoumarin, coumarin and scoparone with detection at a wavelength of 280 nm, while a wavelength of 323 nm proved suitable for esculin, daphnetin, fraxetin, umbelliferone, 4-methylumbelliferone and herniarin. The use of FL detectors, in this case, improved the sensitivity and thus the detection of coumarins exhibiting fluorescence such as esculin, umbelliferone, 4-methylumbelliferone, scoparone and herniarin, especially in the case of detection of umbelliferone and scoparone. Hroboňová et al. [57] examined the number of coumarins such as sculin, daphnetin, fraxetin, umbelliferone, 4-methylumbelliferone, 4-hydroxycoumarin, scoparone, coumarin, herniarin and cinnamyl alcohol in propolis samples for which they developed an appropriate HPLC method. During the development of the method, different mobile phases with different proportions of acetonitrile and acetic acid were tested, with an optimal ratio of acetonitrile content and 0.3% acetic acid in water which was 10:90 (*v*/*v*). The lower amount of acetic acid in the mobile phase (0.1%) during isocratic elution had a negative effect on peak tailing, that is, on the shape and symmetry of the peak, which is improved by increasing the acetic acid content up to 0.3%.

In addition to the FL detector which has improved sensitivity and thus the detection of coumarins exhibiting fluorescence, mass spectrometry in gas and liquid chromatography can also be used to detect coumarin. Raters and Matissek [84] developed a selective and sensitive isotope dilution LC-MS/MS method which was compared with the HPLC-UV method. The developed method showed increased selectivity and sensitivity compared to the HPLC-UV method. That is important since these characteristics of the method are significant in cases of determination of components in food that are not represented in higher concentrations, such as coumarin. Such methods enable the detection of lower concentrations of coumarin in different foods and provide a more realistic picture of the presence of coumarins in the daily diet. Nevertheless, Sproll et al. [19] have developed the HPLC-DAD method which they claim is as suitable for determination and quantification of coumarin in food as the LC-MS/MS method developed by Raters and Matissek [84]. Certainly, the advantage of the HPLC method lies in the simplicity of sample preparation, simpler analysis and processing of results as well as in the lower cost of equipment and labor. According to the authors, in interlaboratory testing, the results obtained are excellent compared to more complex methods, such as LC-MS/MS with deuterium-labeled coumarin as the internal standard.

As mentioned, vanilla extract is one of the most commonly used flavoring ingredients in the diet, including food and beverages. Given the high cost of authentic vanilla extract, artificial vanilla flavor containing synthetically produced vanillin and/or ethyl vanillin is often used, while some manufacturers also add coumarin to enhance the vanilla flavor. Therefore, vanillin, ethyl vanillin and coumarin of twenty-four vanilla extract products on Mexican market was tested using the LC-MS method, as the only chromatographic method to determine the abovementioned components simultaneously in only 13 min and which, in addition, provides a qualitative mass spectral confirmation. The method used was compared with the use of UV detectors, showing that, in most cases, the results obtained were similar, with more accurate results obtained using LC-MS. Such a conclusion is not surprising given the higher level of specificity of LC-MS method. Applying the above method, it has been shown that the addition of coumarin to vanilla products is not common, assuming that such a trend has been influenced by laws and the public [59]. In addition to the above method, the GC method was also used to identify synthetic vanillin as well as other contaminants as early as 1965 [83]. The retention time and peak area were determined to identify the contamination, and the results were shown as relative peak area response, relative to vanillin. Gas chromatography continued to be used to determine contaminants in flavors, and so a simple gas chromatography method in combination with a flame ionization detector was used to detect the prohibited flavors, such as diethylene glycol (DEG), diethylene glycol monoethyl ether (DEGME) and coumarin in food samples. The method developed was validated by determining the limit of detection, linear range, recovery, and reproducibility of the retention time, as soon as it was shown that this method was appropriate for the determination of coumarin in solid and liquid food samples. It is important to note that none of the soft drink and juice tested contain coumarin [73].

Tonka beans, as the first plant from which coumarin was extracted and isolated since they contain 1–3% coumarin, and in rare cases up to 10%, were also investigated. In addition to coumarin, they contain umbelliferone, o-coumaric acid and diterpenoids, as well as the other 138 components that have been detected in the tonka beans extract. The tonka beans extract was analyzed using GC-MS, and then an HPLC method was developed for routine analysis. The following components of tonka beans such as coumarin, o-coumaric acid, 5-hydroxymethylfurfural, melilotic acid, methyl melilotate, ethyl melilotate and dihydrocoumarin in ethanol extract were detected using GC-MS, and then using the HPLC method, these components were detected and quantified not only in ethanol but and the extract obtained by using methanol and acetonitrile as solvent. Accordingly, methanol has been shown to be the most suitable solvent for the extraction of o-coumaric and melilotic acid as well as for coumarin, whose values for extracts obtained under the same conditions are different, with large deviations. A possible reason for this lies in the different and irregular distribution of coumarin crystals within tonka beans. Coumarin values ranged from 1.12–2.33 g/100 g for the extracts obtained with ethanol, methanol and acetonitrile. This method is a simple and convenient method for detecting components in tonka beans extracts [71].

In cases where breast milk is not available or sufficient, the use of the infant formula is necessary for the normal maintenance of infant growth and development. A study conducted in China found that vanillin or coumarin, or sometimes both, are present in some products. Considering that infants are a risk group that needs adequate nutrient intake, without the intake of substances that may have genotoxic and carcinogenic effects, Shen et al. [85] developed and validated a fast and accurate liquid chromatography-quadrupole linear ion trap mass spectrometry method for determination of vanillin, ethyl vanillin, and coumarin. Given the low levels of contaminants in food, conventional analytical techniques are sometimes not sensitive enough to detect and quantify such components. Therefore, more sensitive techniques, such as LC-MS/MS or LC-MS/MS with a triple quadrupole, are increasingly used in the analysis of complex matrices such as food. Given that food is a complex matrix, it is important to keep in mind that matrix effects could influence the error in quantitative LC-MS/MS analysis of food samples. Therefore, Shen et al. [85] added internal standards such as vanillin-13C6 and coumarin-D4 in the sample to correct the influence of the matrix. Similar method, ultra-performance liquid chromatography- linear ion trap triple quadrupole mass spectrometry method (UHPLC-ESI-QqQLIT-MS/MS) was developed, validated and then used to determine coumarin and other phenolics not only in authentic Cinnamomum verum bark samples, but also in samples from south India market. Despite the fact that south India is one of the centers of cinnamon trade, the quality and composition of cinnamon origin from this region has not been tested. In addition, because of the price of cinnamon present on the market, it is very common to mix Cassia cinnamon which has lower-price with Cinnamomum verum, ultimately leading to a high content of coumarin. As for the method itself, applying the UHPLC method over conventional HPLC has led to better resolution in a shorter time. Of the validation parameters tested, it is important to mention the LOD and LOQ given that they were less than 0.36 and 0.81 ng/mL, respectively, which tells us that coumarin can not only be detected but also quantified at very low amounts, which makes this method very sensitive and selective. Of the five cinnamon samples tested from the market, four had high amounts of coumarin, ranging from 819 to 3462 mg/kg, while only one had 19.6 mg/kg. Compared to authentic bark samples containing 12.3 to 143.0 mg/kg, there is a significant difference likely due to mixing with cheaper *C. cassia* and *C. burmanii*. These situations require frequent and constant quality control of cinnamon and cinnamon products on the market, since cinnamon intake with such a high dose of coumarin may exceed the TDI. In addition to the above methods, LC-ESI-QTOF-MS/MS method can be used to identify and determine components, with more accurate quantification including coumarins such as scopolin and scopoletin. The above method was used to determine the composition of Artemisia annua, originally determined by validated HPLC-DAD, which shows efficacy in determining not only coumarin but also other groups of active compounds such as flavonoids and sesquiterpenes [86]. One of the more advanced analytical methods is certainly the UHPLC-QqQ-MS/MS, which was developed to simultaneously identify multiple groups of components including coumarin in Pummelo Fruits. This method, like other chromatographic methods, ensures good separation, selectivity, sensitivity and quantification with respect to good results with validation parameters, but in as short a time as possible, this method takes only 13 min, separating as many as 47 components including isomers [87].

Although sample pretreatment was not used in most of these methods, food, like many natural products, contains many different interfering components, thus making it a complex material that should undergo pre-treatment before chromatographic techniques to ensure reliable results. Considering time-consuming pre-treatments that consume a lot of solvents and loses some of the analytes, it is important to find a pre-treatment that is fast and accurate and. 

Machyňáková et al. [80] therefore propose on-line solid-phase extraction (SPE), as a technique that is versatility and reproducibility, at a lower cost and cost of solvents and human labor, which reduces the error of operation [88,89]. What makes this technique even more compelling is the easy connection between SPE and HPLC using on-line column switching devices, which enables reliable treatment of complex matrices such as food. With regard to sorbents, molecularly imprinted polymers (MIPs) were used in the aforementioned work with respect to stability under different operating conditions and high specificity and selectivity for the desired analyte [90,91]. The reason why they are not used often is the problem of transferring to a column-switching system without loss of extraction selectivity since non-polar solvents and buffers that are not suitable for the conditions of operation of the chromatographic column are often used. In this work, 7-hydroxycoumarin molecularly imprinted polymer was used which proved effective for extracting and separating different coumarins from different sources, such as Cassia cinnamon, chamomile tea and Tokaj specialty wines. The developed method is validated by applying parameters such as LOD and LOQ, linearity, accuracy and precision. According to the results, on-line MISPE-HPLC is a satisfactory method for the determination of coumarin in different food samples, with a simpler sample preparation procedure and faster analysis since the whole process, from sample cleanup to equilibration, takes 13.5 min. However, the efficiency of the sample cleaning process depends on the sample itself, ie its complexity, as well as the accuracy and precision of the results obtained [80].

#### 5.2.2. Spectrophotometric and Spectrofluorimetric Determination

Fluorescence spectroscopy is one of the commonly used methods right because of its selectivity and sensitivity at a low cost. In this area, there is a difference between synchronous fluorescence spectroscopy (SFS) and conventional fluorescence spectroscopy, with the former having several advantages such as simplicity and efficiency in quantitative determination. For this reason, Poláček et al. [58] (developed a rapid, simple and inexpensive method for the determination of coumarin, 4-hydroxycoumarin and dicoumarol in herbal tea (Melilotus Officinalis), whose accuracy and efficacy were then compared with the HPLC method. This synchronous fluorescence method in a combination with PLS multivariate calibration provides excellent results for the calibration and prediction, thus providing a good screening method for herbal tea samples. The spectrofluorimetric method was also used for determination of total coumarins, expressed as scoparone in propolis and propolis products, and the developed method was compared by HPLC with spectrophotometric and fluorescent detection method, by which coumarins from a group of simple coumarins such as esculin, daphnetin, fraxetine, umbelliferone, 4-methylumbelliferone, 4-hydroxycoumarin, scoparone, coumarin, herniarin were determined. In the case of the spectrofluorimetric method, excitation wavelength was set at 370 nm, and the fluorescence intensity was recorded at 423 nm compared to the blank solution. In order to compare the two methods, Hroboňová et al. [57] determined for both methods validation parameters such as the correlation coefficient of the calibration graph, LOD, LOQ, intra-day precision, inter-day precision, and recovery. In both methods, the achieved linear relationship between peak area or fluorescence intensity with the concentration of test components is satisfactory (R^2^ = 0.999), with a wider range of linearity observed by HPLC. On the other hand, fluorescence spectrometry resulted in better LOD and LOQ. However, for both methods, the LOQ for the components is lower than expected for the actual samples, which makes both methods acceptable for simple coumarin determinations. As for the other parameters, intra-day precision was similar for both methods, while better results of inter-day precision were obtained by HPLC. Also, the results for recovery are slightly lower with fluorescence spectrometry, which the authors consider to be the reason for the influence of the matrix. The sample preparation for both methods is similar, while the duration of the test is shorter using fluorescence spectrometry. From all the above it can be concluded that both methods are suitable for the determination of coumarins in real samples. However, by determining total coumarins in propolis samples, 15% higher results were obtained compared to HPLC, with the authors attributing higher matrix effects on the fluorescence of the spectrum [57].

Spectrofluorimetry, together with high-performance liquid chromatography has been tested for the determination of coumarins such as umbelliferone, scopoletin and 4-methylumbelliferone in distilled beverages. As is the problem with most foods, coumarins are also present in very small quantities in distilled beverages, making it difficult to detect and quantify them using common analytical techniques. In the work of Fernández Izquierdo et al. [56], they compared in detail two analytical methods using different statistical methods, spectrofluorometry and high-performance liquid chromatography, both of which show the possibility of application in the determination of coumarin in distilled beverages, with certain differences, especially in accuracy and precision. In addition, the authors suggested that by modifying the HPLC method, even better accuracy and precision can be achieved, and given that the method is faster and simpler, it often has the advantage of determining coumarin. However, analysts can choose between the two methods given that they are both sensitive enough and give accurate and accurate results for umbelliferone, scopoletin and 4-methylumbelliferone in distilled beverages.

Vanilla extracts, in addition to chromatographic methods, were also investigated using Fourier transform mid-infrared (MID-FTIR) spectroscopy coupled with chemometric analysis. This method, compared to the chromatographic methods that are more expensive and require longer sample preparation, is simple and fast, without the use of reagents and pre-treatment. For this reason, the aim of Moreno-Ley et al. [92] was to conduct an analysis of the content of coumarin and ethyl vanillin in vanilla extracts using the MID-FTIR spectroscopy and the HPLC-DAD method to design chemometric models that can then be applied to commercial samples. Samples that were adulterated were tested, ie to which a certain amount of ethyl vanillin and coumarin were added and commercially available vanilla extracts, and a predictive model was thus constructed using several algorithms, the best of which was partial least squares with single y-variables. Although the predictions are better for ethyl vanillin compared to coumarin, given that the spectrum of samples forged with coumarin is similar to the control sample, with the above algorithm ethyl vanillin and coumarin were determined in amounts of 0.20–10% and 0.1–10 ppm, respectively. Given that there were no significant differences between the developed model and the HPLC analysis, as well as very small detectable quantities, the developed method and model are a viable method, especially in conditions where results are required immediately [92].

A similar method involving the use of fluorescence spectrometry, and the chemometric approach, as well as extraction using a molecularly imprinted solid-phase was also used to analyze coumarin in wine. Unlike the work of Machyňáaková et al. [80], MIP SPE used here was offline type, used as well as online to clean and prepare samples for analysis. Samples were first analyzed using a validated HPLC method with UV–VIS and fluorescence detectors to determine coumarin content such as esculin, coumarin, herniarin, 4-methylumbelliferone, scoparone, scopoletin, and then analyzed using fluorescence spectrometry. Data collected from both analyzes, such as coumarin concentrations in the samples as well as emission spectral data and synchronous fluorescence spectra, were then used to develop the PLS model. Comparing the model developed with the results obtained by HPLC, a good correlation is observed, which supports the above method and model for further use in the analysis of coumarins in wine [93].

**Table 6:** Overview of newer methods of detection and quantification of coumarin and related components.

Sample	Method	Work Conditions	Detected Compounds	Literature
Cinnamons and cinnamon containing foods	HPTLC	HPTLC plates silica gel 60 mixture of n-hexane—ethyl acetate—ammonia (3.8:1.3:0.05, *v*/*v*/*v*) 10% ethanolic KOH solution for detection 10% methanolic PEG 400 solution - stabilization of the fluorescence 366 nm	coumarin	[17]
	HPTLC-MS	100% methanol 0.1 mL min^−1^ capillary and source gas temperature 250 °C capillary voltage 150 V source voltage offset 10 V 100–600 *m*/*z*		
Cinnamon-containing food products	HPLC-DAD	5 mM ammonium acetate buffer, 0.2% acetic acid: ACN/MeOH (1:2) gradient elution 0.2 mL min^−1^ 40 °C 279.8 nm	coumarin	[19]
Distilled beverages	HPLC-FL	3% glacial acetic acid in water:ACN with 3% glacial acetic acid gradient elution 1 mL min^−1^ room temp. excitation and emission 340 and 425 nm	umbelliferone, scopoletin and 4-methylumbelliferone	[56]
	Spectrofluorometry	excitation and emission wavelengths 340 and 425 nm 25 ± 1 °C slits set at an aperture of 3.0 nm		
Propolis	Fluorescence spectrometry	excitation and emission slits 5 nm fluorescence emission spectra from 380 to 600 nm excitation wavelength 370 nm emission spectrum at 23 °C	sculin, daphnetin, fraxetin, umbelliferone, 4-methylumbelliferone, 4-hydroxycoumarin, scoparone, coumarin, herniarin and cinnamyl alcohol	[57]
	HPLC	ACN with 0.3% acetic acid:ACN gradient elution 1 mL min^−1^ 23 °C 280 and 323 nm		
Herbal tea from sweet clover plant (*Melilotus officinalis*)	SFS	excitation and emission splits 5 nm scan speed 200 nm/min PMT Voltage 500 Volts the excitation wavelength range at 200–400 nm Δλ = 90 nm	coumarin, 4-hydroxycoumarin and dicoumarol	[58]
	HPLC	0.3% acetic acid in methanol: 0.3% acetic acid in water gradient method 0.5 mL min^−1^ 23 °C 280 nm		
Vanilla extract products	LC-UV-MS	ACN: 0.1% formic acid (35:65) isocratic elution 0.25 mL min^−1^ 20 °C 254 nm quantification was based on a peak area ratio of the SIM signals of the analyte and the IS	coumarin, vanillin and ethly vanillin	[59]
Cinnamon-containing food products	UHPLC-DAD	5% MeOH in demineralized water/ACN Gradient elution 0.6 mL min^−1^, 45 °C 278.1 nm	coumarin	[64]
Cinnamon samples	UHPLC-ESI-QqQLIT-MS/MS	0.1% formic acid in water:ACN gradient elution 0.3 mL min^−1^ 30 °C both positive and negative ESI modes Source temperature 550°C range of *m*/*z* 100–1000	coumarin, scopoletin, *o*-coumaric acid, cinnamic acid and cinnamaldehyde	[65]
The seeds of *Dipteryx odorata* (tonka beans)	HPLC	0.0001% 85% *o*-phosphoric acid:ACN:MeOH gradient elution 1 mL min^−1^ 45 °C 260–275 nm	coumarin, *o*-coumaric acid, 5-hydroxymethylfurfural, melilotic acid, methyl melilotate and ethyl melilotate and dihydrocoumarin	[71]
	GC-MS	Helium 70–280 °C 70 eV 0.2 mA mass range *m*/*z* 50–380		
Cassia Bark (*Cortex Cinnamomi*)	HPLC quantitative analyses	ACN:0.04% acetic acid (25:75) isocratic elution 1 mL min^−1^ 20 °C 250, 280 nm	cinnamaldehyde, cinnamic acid, coumarin and cinnamyl alcohol	[72]
	HPLC fingerprint analysis	ACN:0.02% acetic acid gradient elution 1 mL min^−1^ 20 °C 280 nm	coumarin, cinnamic acid, cinnamaldehyde, unknown, and eugenol	
Food containing soft drinks and juice, infant formula and food, cereals, flours and snacks	GC-FID	Oxygen free nitrogen 1 mL min^−1^ 100–260 °C	diethylene glycol, diethylene glycol monoethyl ether, coumarin and caffeine	[73]
Food flavored with vanilla	TLC	Ethyl acetate, Chloroform, Propanol, Acetic acid, Hexane gradient elution AMD chamber 280 nm—densitometer	vanillin, ethyl vanillin, 4-hydroxy-benzaldehyde, 4-hydroxybenzoic acid, 4-hydroxybenzyl alcohol, vanillic acid, coumarin, piperonal, anisic acid, and anisaldehyde	[74]
*Cinnamomum cassia* Blume	HPLC	ACN:0.5% acetic acid in water (25:75, *v*/*v*) isocratic elution 1.0 mL min^−1^ 25 °C detection at 278 nm	Coumarin	[76]
Spice/spice mixture; Cinnamon cookies; Gingerbread	HPLC–UV	Ammonium acetate (5 mmol/L)/ACN:MeOH (1:2) gradient elution 0.8 mL min^−1^ 40 °C 279 nm	coumarin	[78]
	LC–MS/MS	MeOH:ACN:0.1% formic acid (80:0.1:19.9) isocratic elution 0.25 mL min^−1^ 20 °C identified by selected-reaction monitoring (SRM) positive electrospray ionization mode (ESI+)		
Cassia cinnamon, chamomile tea	HPLC	ACN: 0.3% acetic acid gradient elution 1 mL min^−1^ 20 µL 30 °C 280 and 323 nm	6,7-dihydroxycoumarin 7,8-dihydroxy-6-methoxy-coumarin 7-hydroxycoumarin 7-hydroxy-4-methylcoumarin 6,7-dimethoxycoumarin coumarin 7-methoxycoumarin	[80]
	On-line MISPE-HPLC system	20 mg of MIP sorbent switching sstem washing with H_2_O HPLC conditions as above		
Infant formula	LC-QqLIT-MS	0.1% formic acid/ACN gradient elution 0.25 mL min^−1^ 30 °C ESI positive mode Source temperature 500°C Ionization voltage 5000 V	vanillin, ethyl vanillin, and coumarin	[85]
*Artemisia annua*	HPLC-DAD	0.1% formic acid:ACN gradient elution 0.6 mL min^−1^ 30 °C 10 µL 210 and 360 nm	rutin, cynaroside, isorhamnetin, chrysosplenol D and casticin, scopolin and scopoletin, arteannuinB, artemisinin, dihydroartemisinic acid and artemisinic acid	[86]
	LC-ESI-QTOF-MS/MS	positive and negative mode 50 to 1000 Da 180°C the end plate offset was−500V capillary voltages were 4500 V and −3500 V		
Pummelo Fruits	UHPLC-QqQ-MS/MS	0.1% formic acid:MeOH gradient elution 0.3 mL min^−1^ 2 µL 40 °C positive and negative modes curtain gas 20.0; ionspray voltage (IS) ±4500.0 temperature (TEM) 500.0; ion source gas 1 (GS1) 50.0; ion source gas 2 (GS2) 50.0	47 components, 12 coumarins and furocoumarins; umbelliferone, scoparone, psoralen, bergaptol, xanthotoxin, Limettin, bergapten, isomeranzin, 6′,7′dihydroxybergamottin, imperatorin, isoimperatorin, 6′,7′epoxybergamottin	[87]
Cassia cinnamon, chamomile tea	HPLC	ACN: 0.3% acetic acid gradient elution 1 mL min^−1^ 20 µL 30 °C 280 and 323 nm	6,7-dihydroxycoumarin 7,8-dihydroxy-6-methoxy-coumarin 7-hydroxycoumarin 7-hydroxy-4-methylcoumarin 6,7-dimethoxycoumarin coumarin 7-methoxycoumarin	[80]
	On-line MISPE-HPLC system	20 mg of MIP sorbent switching sstem washing with H_2_O HPLC conditions as above		
Vanilla extracts	MID-FTIR spectroscopy	range of 4000–550 cm^−1^ resolution of 4 cm^−1^ 64 scans	ethyl vanillin, coumarin	[92]
	HPLC-DAD	ACN: acid water (pH 2.3) gradient elution 30 °C 1µL 230, 260, 280 nm		
Tokaj wine	HPLC-DAD HPLC-FL	MeOH/acetic acid:1% of acetic acid gradient elution 23 °C 20 µL 280, 320, 450 nm	esculin, coumarin, herniarin, 4-methylumbelliferon, scoparone, scopoletin	[93]
	fluorescence spectroscopy	excitation and emission slit width 5 nm excitation wavelength 320 nm synchronous fluorescence spectra → 250–400 nm range		

TLC—thin-layer chromatography; HPLC—high-performance liquid chromatography; UHPLC—ultra high-performance liquid chromatography; GC-MS—gas chromatography–mass spectrometry; FL—fluorescence detector; LC-UV-MS—liquid chromatographyultraviolet detection-mass spectrometry; DAD—diode-array detection; UHPLC-MS—ultra high-performance liquid chromatography-mass spectrometry; LC-MS—liquid chromatography–mass spectrometry; LC-MS/MS—liquid chromatography/tandem mass spectrometry; GC-FID—gas chromatography – flame ionization detector; SFS—synchronous fluorescence spectroscopy; LC-QqLIT-MS—liquid chromatography-quadrupole linear ion trap mass spectrometry; UHPLC-ESI-QqQLIT-MS/MS—ultra-performance liquid chromatography- linear ion trap triple quadrupole mass spectrometry; UHPLC-QqQ-MS/MS—ultra high-performance liquid chromatography coupled with triple quadrupole mass spectrometry; MISPE-HPLC—molecularly imprinted solid-phase extraction- high-performance liquid chromatography; MID-FTIR—Fourier transform mid-infrared spectroscopy.

## 6. Conclusions

Recently, scientists have increasingly investigated the health security of coumarins in food. They are developing the ideal methods with better analytical characteristics, which may be applied to the complex foods matrices and may be accommodated to suit the necessities of a certain coumarin.

Earlier studies on rats, which were fed with very large doses of coumarin over long periods, discovered that coumarin can have hepatotoxic effects. These hepatotoxic effects are not yet clearly confirmed in humans, but it was revealed that different metabolism modes of coumarin in humans compared to rodents. However, evidence of coumarin hepatotoxicity has to be considered when discussing the health security of coumarin intake.

Although cinnamon may have beneficial effects on human health, a large amount of cinnamon intake may have negative impacts, since cinnamon contains high levels of coumarin itself. Therefore, EFSA prescribed the TDI value which defines the amount of coumarin that a person can ingest daily over a lifetime without appreciable health risk. The TDI is 0.1 mg of coumarin per kg of body weight daily. Some research has shown that by consuming a large amount of food containing Cassia cinnamon, it is possible to surpass the TDI of coumarin by eating that food alone, especially if large amounts of such foods are eaten every day. Parents need to be extremely careful with their children because they consume more sweet food containing cinnamon than adults. Considering those, it is necessary to reduce the intake of Cassia cinnamon to avoid health risk. In that context, it is necessary to work on a consumers’ better understanding of consumption risks of the coumarin-containing food. It could be accomplished by indicating the cinnamon type on the products’ packages, or giving some official advice on the moderate coumarin consumption, especially for sensitive groups of people such as children. Moreover, it is also important to provide better knowledge to the manufacturers about EU regulations in terms of coumarin, in order to avoid the production of cinnamon based products without exceeding the EU limits for coumarin.

## Figures and Tables

**Table 1:** Compound food in which the presence of the coumarin is restricted [27].

Compound Food	Maximum Level of Coumarin (mg/kg)
Traditional and/or seasonal bakery wares containing a reference to cinnamon in the labeling	50
Breakfast cereals including muesli	20
Fine bakery ware, with the exception of traditional and/or seasonal bakery wares containing a reference to cinnamon in the labeling	15
Desserts	5
